# Associations of preoperative anemia and postoperative hemoglobin values with hospital costs in total knee arthroplasty (TKA)

**DOI:** 10.1007/s00402-023-04929-4

**Published:** 2023-06-12

**Authors:** Caroline Schatz, Werner Plötz, Johannes Beckmann, Katharina Bredow, Reiner Leidl, Peter Buschner

**Affiliations:** 1 Ludwig-Maximilians-Universität München, LMU Munich School of Management, Institute for Health Economics and Health Care Management, Ludwigstr. 28, 80539 Munich, Germany; 2 Helmholtz Zentrum München, Institute for Health Economics and Health Care Management, Munich, Germany; 3 Environmental Health Center at Helmholtz Munich, Munich, Germany; 4 Krankenhaus Barmherzige Brüder München, Akademisches Lehrkrankenhaus der Technischen Universität München, Munich, Germany; 5 Klinikum rechts der Isar, Technical University Munich, Munich, Germany; 6 Orthopaedic Praxis Munich-Nymphenburg, Munich, Germany

**Keywords:** Osteoarthritis, Joint replacement, Cost, Hemoglobin, Anemia, Knee

## Abstract

**Background:**

Total knee arthroplasty are among the most frequently conducted surgeries, due to an aging society. Since hospital costs are subsequently rising, adequate preparation of patients and reimbursement becomes more and more important. Recent literature revealed anemia as a risk factor for enhanced length of stay (LOS) and complications. This study analyzed whether preoperative hemoglobin (Hb) and postoperative Hb were associated with total hospital costs and general ward costs.

**Methods:**

The study comprised 367 patients from a single high-volume hospital in Germany. Hospital costs were calculated with standardized cost accounting methods. Generalized linear models were applied to account for confounders, such as age, comorbidities, body mass index, insurance status, health-related quality of life, implant types, incision-suture-time and tranexamic acid.

**Results:**

Preoperative anemic women had 426 Euros higher general ward costs (p < 0.01), due to increased LOS. For men, 1 g/dl less Hb loss between the preoperative value and the value before discharge reduced total costs by 292 Euros (p < 0.001) and 161 Euros fewer general ward costs (p < 0.001). Total hospital costs were reduced by 144 Euros with 1 g/dl higher Hb on day 2 postoperatively for women (p < 0.01).

**Conclusion:**

Preoperative anemia was associated with increased general ward costs for women and Hb loss with decreasing total hospital costs for men and women. Cost containment, especially reduced utilization of the general ward, may be feasible with the correction of anemia for women. Postoperative Hb values may be a factor for adjustments of reimbursement systems.

**Level of evidence:**

Retrospective cohort study, III.

**Supplementary Information:**

The online version contains supplementary material available at 10.1007/s00402-023-04929-4.

## Background

End-stage osteoarthritis (OA) is a global burden with a high prevalence, due to an aging society, with total knee arthroplasty (TKA) as a common surgery to improve pain and function [16]. The problem of rising costs for healthcare systems entails the challenge of preparing patients appropriately and designing adequate reimbursement systems. Recently, comorbidities were investigated as risk factors for high costs, especially for patients who exceeded the bundled-payment system for total joint replacements (TJR) in the US [15, 39]. One of the possible modifiable comorbidities was preoperative anemia, which increased the total episode of care costs [15, 39]. Patients with revision total hip arthroplasty and iron deficiency anemia had higher length of stay (LOS), exceeded costs in the bundled-payment system and more complications compared to patients without anemia [32]. For primary total hip arthroplasty (THA), anemic patients below 11 g/dl Hb had higher LOS and below 12 g/dl further risk assessments were recommended [30]. The total episode of care costs in the bundled payment system includes all costs, until 90 days after discharge from the hospital [21]. Hence, risk adjusted pricing was recommended, because without adequate adjustments special patient groups may lose access to TJR [21]. Hospitals in the US had higher charges on the day of surgery, readmissions and emergency visits for patients with THA and preoperative iron deficiency anemia [29]. Similarly, poorer outcomes were found for anemic TKA patients [19]. Utilizing a cost simulation model for THA and TKA, a Swedish study found increased costs for nursing days and reduced transfusion costs during the hospital stay for patients with preoperative correction of iron deficiency anemia [37]. Patients who attended a blood management program had lower risk for blood transfusions, postoperative complications, and LOS for primary arthroplasties [1]. However, just a part of the anemic patients, who attended the blood management program, were no longer anemic at the day of surgery [1]. This shows that preoperative anemia may be corrected partly, but not for all patients undergoing TKA. Guidelines about preoperative anemia are currently under consideration in Germany and no clear recommendations for preparing anemic patients exist [7]. The increased LOS and higher complication rates for those patients indicate higher resource utilization and thereof higher hospital costs. LOS is especially relevant for nursing and the general ward, because the longer the patients need care, the higher the costs. Last, but clearly not least, preoperative as well as postoperative anemia is associated with increased morbidity, mortality [24] and infection rate [26].

Due to the challenging correction of anemia preoperatively, the correction of iron deficiency anemia is also done postoperatively, but the improvements were not immediately significant during the hospital stay [18]. Additionally, postoperative moderate and severe anemia was associated with preoperative Hb values and intraoperative blood loss for TKA [4]. Preoperative Hb for men < 13.85 g/dl and < 13.15 g/dl for women resulted in moderate or severe anemia, postoperatively [4]. Additionally, to the treatment of iron deficiency anemia with iron supplements, tranexamic acid (TRX) is applied intraoperatively, because it is regarded to be effective for reducing blood loss and transfusion rates for THR and TKA [2, 12]. Due to the reduced transfusion rates with TRX the preoperative screening of patients with TJA was discussed controversially [34, 35].

Since Hb values are important screening parameters, preoperatively and postoperatively, this study investigated their association to hospital costs, in order to bridge the gap between medical care and financial management. The preoperative Hb values were examined, to reveal whether anemia is associated with not only negative effects, such as complications and increased LOS for the patient, but also whether its economic impact may provide a link to cost containment. Associations of preoperative anemia and costs might be an argument for standardized correction of anemia to reduce costs for the healthcare system with simultaneous positive effects for the patients. Although, there may be patient groups where a correction is not feasible, as mentioned implicitly in the discussion about preoperative anemia in the bundled-payment system, which revealed the research gap of postoperative Hb values and their use for risk adjustments into reimbursement systems. In this study the hospital costs were calculated for the reimbursement system of diagnoses related groups (DRG) in Germany. These hospital costs included inpatient costs and were sub-divided into general ward costs, which allowed a detailed analyses of cost components, especially with regard to LOS. An association of these hospital costs with postoperative Hb values may be the basis for risk adjustments in reimbursement systems. To the best of our knowledge this is the first paper to investigate preoperative anemia and its association with hospital costs for TKA as well as the course of postoperative Hb values and their association with hospital costs. In the last decade, transfusion rates were reduced sharply, and surgical techniques were improved, due to TRX. Hence, only patients without transfusions were analyzed to explore whether preoperative and postoperative Hb values were associated with hospital costs. The following research (RQ) questions were developed:

RQ1: Was preoperative anemia associated with increased total hospital costs and general ward costs for TKA?

RQ2: Which postoperative Hb values were associated with total hospital costs and general ward costs for TKA?

## Materials and methods

The observational study comprised 367 patients from a single hospital in Germany, which was certified as an arthroplasty center of maximum medical care (endoCert^®^). The study is a part of the research project Munich Network Health Care Research—MobilE-Net. Patients with primary TKA, due to OA, were retrospectively included. All patients had one TKA, no simultaneous bilateral TKA. This study was approved by the ethics committee of Ludwig-Maximilians-Universität München (reference number: 18–274). All patients had a surgery between 5 August 2019 and 30 November 2020 and signed the consent to participate. The cross-sectional data was split into men and women, because Hb values differ between genders.

### Surgical procedure and bleeding management

Anemia screening was performed prior to surgery to rule out preoperative anemia. In case of values below 12 g/dl, a preoperative clarification was performed by the resident physician or specialist and, if possible, treatment was given. Especially in case of iron deficiency anemia, intravenous iron substitution was performed before surgery. Intraoperatively, tranexamic acid was administered intravenously and/or locally, provided there were no contraindications (e.g., condition after thromboembolic events, coronary heart disease and condition after stenting, epilepsy, etc.). Tourniquets or drains were not used.

Thromboprophylaxis was performed in all cases with low-molecular-weight heparin from the evening of the day of surgery for 11–14 days postoperatively according to the applicable guideline. In cases with an increased thromboembolic risk, semi-therapeutic doses were administered from the 3rd postoperative day. In exceptional cases (for example, in the presence of allergies or injection phobias), new oral anticoagulants (NOACs) were also used.

About 10% of patients were on antiplatelet medication (usually aspirin). In patients taking warfarin or other oral anticoagulants prior to surgery, the medication was paused preoperatively and bridged with a low molecular weight heparin if necessary.

TRX was used according to an in-house standard in all cases in which there was no contraindication to its administration, regardless of the presence of preoperative anemia, except patients had a thrombosis, deep vein thrombosis or pulmonary embolism. Additionally, TRX was not given to patients with an apoplex, cardiac infarction or stent implantation within 12 months before surgery.

### Hospital costs

Hospital costs were collected for each patient from the financial department of the hospital and were calculated according to the standard of the Institute for the Hospital Remuneration System in Germany [36]. Total hospital costs comprised all inpatient costs. This included direct attributable costs, such as implants and drugs, but also indirect, non-attributable costs, such as general ward costs and the cost for the surgery. Outpatient costs, such as preoperative physician costs, or costs for rehabilitation were not included. 0.5% inflation rate was considered for patients from 2019.

### Preoperative anemia and Hb values

Preoperative anemia was included as a binary variable with one indicating anemia and zero otherwise. The definition of anemia follows the suggestions of the World Health Organization (WHO) [38], for men < 13 g/dl and for women < 12 g/dl. There were no patients with a severe preoperative anemia, according to the WHO definition (< 8 g/dl for men and women). Hb values were measured for each patient preoperatively, at the first and second day after the surgery and on the day before discharge. Additionally, the Hb loss as the difference between the preoperative Hb and the Hb on the day before discharge was calculated.

### Confounders

Confounders were selected thoroughly, because the cross-sectional, observational data were not randomized. The statistical analyses were based on a regression model, because with univariate statistical tests confounding is not possible. Hence, the study evaluated several variables as confounders, with reasons.

General health before surgery was measured with the EQ VAS (EuroQoL Visual Analog Scale), part of the EQ-5D-5L questionnaire [3]. This confounder served as a proxy for the health status before surgery. This health status was considered relevant, because patients with preoperative anemia might have a lower health status than patients without anemia. Hence, this circumstance was considered with the confounder of the preoperative EQ VAS. Additionally, age and body mass index (BMI) were collected. The age was considered as a relevant confounder, because with the age of patients the costs may vary, due to possible higher care needs of elderly patients. Similarly, for patients with a high BMI, the care needs might be higher as for patients with a normal BMI. As a proxy for the socioeconomic status the insurance status was evaluated, distinguishing between statutory and non-statutory insurance, because Edwards et al. [9] found associations of socioeconomic status and utilization of THA. To control for comorbidities, the ASA Score (American Society of Anesthesiologists Physical Status Classification System) [27] was included as a measure of the anesthetic assessment. Furthermore, Charlson Scores [5] and Elixhauser Scores [11], adapted with weightings for orthopedics from Menendez [22], were calculated from orthopedics’ notes. Patients were categorized into 3 groups, with ascending comorbidities (< 0, = 0, > 0). Comorbidities were considered to be relevant confounders, because patients with a higher comorbidity score might be more costly than patients with a low comorbidity score. Hence, the ASA Score and the Menendez Score were included in the regression model to control for this circumstance. Intraarticular TRX was included as a binary confounder, because TRX reduces generally blood loss during the surgery. Patients with TRX were considered to have a more stable Hb value postoperatively than patients without TRX. The type of implant was previously investigated as a cost driver for joint replacements [31]. Hence, the implant types were included as categorical variables, distinguished between unicompartmental and bicondylar, with and without patella, and constrained versus unconstrained.

Since the study was conducted during the COVID-19 pandemic, the lockdown status was included [28]. The lockdownstatus separated the patients into three groups, namely into patients with a surgery before the first lockdown, during the first lockdown and after the first lockdown, in Germany. This confounder was considered relevant, because during the first lockdown elective surgeries were sharply reduced. Additionally, hospital costs were affected by the COVID-19 pandemic, resulting in higher costs during and after the first lockdown in Germany [28].

### Sample corrections

From the 367 patients, 1 patient with 0 Euros total costs, 1 patient with 0 Euros implant costs, 1 patient with 0 days of LOS and 1 patient with a BMI > 200 were excluded, because all these values seemed to be not meaningful. Outliers were detected using boxplots and excluded above and below 1.5 times the 75% quantiles (22 patients). 341 patients remained for the analyses.

### Statistical analyses

Generalized linear models (GLM) with gamma distribution and log-link function were applied to account for confounders, due to the positive values of hospital costs. Coefficients below 1 indicate a negative association with the respective costs and coefficients above 1 indicate a positive association. The coefficients were interpreted in percentages of the mean. Anemia and each of the 4 Hb values were included in the models with the same confounders for men and women, referring to total- and ward costs respectively, resulting in 20 models. Model selection was done with the Akaike information criterion (AIC) [8] and the model with the lowest AIC was reported in the results. The pseudo R^2^ from McFadden was used to evaluate the fit of the models, with a R^2^ between 0.2 and 0.4 indicating an excellent fit [20]. The minimum threshold for p-values was 0.05. Multicollinearity was detected with the variation inflation factor and a threshold below 2.5 was considered unproblematic [14]. The data collection was done with Microsoft Excel^®^ and the statistical analyses with R [13, 25, 33, 40–42].

### Sensitivity analyses

Several sensitivity analyses were performed [23]. The main results did not change. Details were reported in the Supplementary material A.

## Results

### Study cohort

The descriptive results show that 147 men and 194 women received TKA with 21 preoperative anemic men and 21 preoperative anemic women. Total hospital costs were 8119 Euros for men with preoperative anemia and 7859 Euros for men without preoperative anemia. For women, the total hospital costs were 8204 Euros with preoperative anemia and 8015 Euros without preoperative anemia. Total hospital costs for these patients were about 260 Euros higher for men and 189 Euros higher for women with preoperative anemia and total hospital costs were generally higher for women compared to men. Normal ward costs were also increased for patients with preoperative anemia (331 Euros for men, 376 Euros for women). The mean Hb values comprised 11.9 g/dl for men with preoperative anemia and 11.4 g/dl for preoperative anemic women. For patients without preoperative anemia the mean Hb were 14.9 g/dl for men and 13.7 g/dl for women. The course of Hb was similar for both genders, with − 1.8 g/dl for men with preoperative anemia and − 1.6 g/dl for women (without preoperative anemia − 2.8 g/dl for men and − 2.6 g/dl for women). The LOS was increased by 1 day for anemic women, but only by a third of a day for men. TRX was applied by roughly 78% of all patients with very low costs, because the drug costs per patient amounted in approximately 75 Euros. The implant costs were a major cost component with approximately 2000 Euros per patient (Table 1).

**Table 1:** Study cohort

	Entire cohort (N = 341)			Men (N = 147)						Women (N = 194)					
				Preoperative anemia^a^ (N = 21)			No preoperative anemia (N = 126)			Preoperative anemia^a^ (N = 21)			No preoperative anemia (N = 173)		
	n	Mean ± SD	Min|max	n	Mean ± SD	Min|max	n	Mean ± SD	Min|max	n	Mean ± SD	Min|max	n	Mean ± SD	Min|max
		(%)			(%)			(%)			(%)			(%)	
Hospital costs per patient	341	7976 ± 1008	5799|10952	21	8119 ± 1190	5799|10702	126	7859 ± 1036	6144|10598	21	8204 ± 950	6748|10555	173	8015 ± 869	6211|10952
General ward costs per patient	341	2954 ± 670	1140|5137	21	3116 ± 874	1303|4678	126	2785 ± 664	1140|4734	21	3381 ± 741	2271|5137	173	3005 ± 606	1635|5000
Implant costs per patient	341	2020 ± 463	1503|4528	21	2070 ± 518	1503|3609	126	2014 ± 495	1617|4518	21	1891 ± 198	1645|2481	173	1920 ± 455	1645|4528
Drug costs per patient	341	75 ± 14	48|144	21	79 ± 16	48|121	126	73 ± 13	48|126	21	80.0 ± 13.4	58.7|121.5	173	76.4 ± 14.0	51.1|144.4
Hemoglobin values															
Preoperative	341	13.9 ± 1.4	9.6|19.0	21	11.9 ± 0.9	9.6|12.8	126	14.9 ± 1.1	13.0|19.0	21	11.4 ± 0.5	10.0|11.9	173	13.7 ± 0.9	12.0|15.8
Day 1 postoperative	341	11.6 ± 1.4	8.1|15.3	21	10.1 ± 1.0	8.6|12.7	126	12.6 ± 1.3	8.6|15.3	21	10.1 ± 1.0	8.1|12.2	173	11.2 ± 1.1	8.6|13.8
Day 2 postoperative	341	11.4 ± 1.5	7.9|15.7	21	10.2 ± 1.3	8.1|12.7	126	12.3 ± 1.4	8.9|15.7	21	9.9 ± 1.1	7.9|12.0	173	11.1 ± 1.3	8.1|14.2
Discharge	341	11.3 ± 1.5	6.8|14.8	21	10.1 ± 1.1	7.6|12.3	126	12.1 ± 1.5	7.7|14.8	21	9.8 ± 1.5	6.8|13.1	173	11.0 ± 1.2	8.2|14.1
Hemoglobin loss (discharge-preoperative)	341	− 2.6 ± 1.2	− 7.2|1.3	21	− 1.8 ± 0.9	− 3.2|0.1	126	− 2.8 ± 1.3	− 7.2|− 0.4	21	− 1.6 ± 1.4	− 4.3|1.3	173	− 2.6 ± 1.1	− 4.9|0.5
Implant types															
Unicompartmental	62	(19)		1	(4)		32	(25)		5	(24)		24	(14)	
Bicondylar without patella, not rotational	144	(42)		9	(42)		56	(44)		8	(38)		71	(41)	
Bicondylar with patella, not rotational	127	(37)		10	(48)		36	(29)		8	(38)		73	(42)	
Bicondylar without patella, rotational	3	(1)		1	(4)		0	(0)		0	(0)		2	(1)	
Bicondylar with patella, rotational	5	(1)		0	(0)		2	(2)		0	(0)		3	(2)	
Length of stay	341	7.3 ± 1.7	3.0|14.0		7.4 ± 2.5	3.0|14.0		7.1 ± 1.9	3.0|14.0		8.3 ± 1.5	5.0|12.0		7.4 ± 1.4	3.0|11.0
Incision-suture time	341	62.9 ± 17.2	35|155		62.7 ± 13.1	42|90		66.8 ± 19.4	38|155		60.8 ± 17.4	41|109		60.2 ± 15.6	35|121
Tranexamic acid intraarticular	268	(78)		19	(90)		96	(76)		15	(71)		138	(80)	
EQ VAS	341	57.5 ± 22.3	0|100		62.4 ± 21.5	3|90		59.7 ± 23.1	0|97		57.6 ± 25.9	0|100		55.2 ± 21.3	0|100
Age in years	341	71.3 ± 9.0	42|87		76.8 ± 4.6	67|84		71.1 ± 8.7	52|87		73.1 ± 11.3	42|87		70.6 ± 9.2	44|87
BMI	341	29.0 ± 5.9	18|58		26.7 ± 4.6	19.9|39.8		28.4 ± 4.6	19.0|44.8		28.3 ± 5.9	20.6|40.6		29.8 ± 6.8	17.7|57.8
Insurance status non-statutory	40	(12)		6	(29)		15	(12)		2	(10)		17	(10)	
ASA score	341	2.1 ± 0.5	1|4		2.4 ± 0.6	1|3		2.1 ± 0.5	1|4		2.3 ± 0.5	2|3		2.1 ± 0.4	1|3
1	21	(6)		1	(4)		10	(8)		0	(0)		10	(6)	
2	250	(73)		10	(48)		88	(70)		14	(67)		138	(80)	
3/4	70	(21)		10	(48)		28	(22)		7	(33)		25	(14)	
Menendez score	341	− 1.3 ± 3.2	− 9|12		0.4 ± 2.9	− 4|7		− 0.6 ± 3.1	− 9|8		− 1.3 ± 3.9	− 8|12		− 2.0 ± 3.1	− 9|12
< 0	180	(53)		6	(29)		54	(43)	12		(57)		108	(62)	
= 0	93	(27)		8	(38)		40	(32)	6		(29)		39	(23)	
> 0	68	(20)		7	(33)		32	(25)	3		(14)		26	(15)	
Lockdownstatus															
Before first COVID-19 lockdown	154	(45)		10	(48)		61	(48)	10		(48)		73	(42)	
During first COVID-19 lockdown	30	(9)		2	(10)		13	(10)	4		(19)		11	(6)	
After first COVID-19 lockdown	157	(46)		9	(42)		52	(42)	7		(33)		89	(52)	

^a^According to WHO < 12 g/dl for women and < 13 g/dl for men; min = minimum; max = maximum; SD = standard deviation; mean = arithmetic mean, costs in Euros

### Results preoperative anemia

When accounting for confounders, the results for preoperative anemia revealed different findings for men and women. Preoperative anemia increased general ward costs per patient for women by 14.1% compared to patients without anemia, in total approximately 426 Euros. General ward costs for men did not indicate any differences for anemic versus non-anemic patients. Similarly, total costs were not significantly different for preoperative anemic women and preoperative anemic men. Implant types and TRX were significantly associated with general ward costs and total costs for men and women. Whereas incision-suture time was associated with total costs for men and women, but not for general ward costs. The pseudo R^2^ indicated a relatively good fit for all models (Table 2).

**Table 2:** Results preoperative anemia

Men					Women				
General ward costs per patient	Coefficient	CI 95%	p-Value		General ward costs per patient	Coefficient	CI 95%	p-Value	
Intercept	0.915	536|1566	< 0.001	***	Intercept	1645	1134|2387	< 0.001	***
Preoperative anemia	0.986	0.884|1.102	0.803	N.S.	Preoperative anemia	1.141	1.044|1.250	0.005	**
EQ-VAS	1.000	0.999|1.002	0.654	N.S.	EQ-VAS	0.999	0.997|1.000	0.016	*
Age	1.007	1.003|1.012	0.003	**	Age	1.000	1.000|1.007	0.023	*
BMI	1.004	0.995|1.015	0.337	N.S.	BMI	1.000	0.999|1.009	0.100	N.S.
Insurance status	0.976	0.875|1.091	0.666	N.S.	Insurance status	1.057	0.962|1.164	0.252	N.S.
ASA Score	1.054	0.969|1.137	0.221	N.S.	ASA Score	1.032	0.961|1.108	0.387	N.S.
Menendez Index	1.036	0.986|1.089	0.164	N.S.	Menendez Index	1.028	0.990|1.068	0.161	N.S.
TRX ia	1.176	1.074|1.285	< 0.001	***	TRX ia	1.116	1.043|1.193	0.002	**
Implant types	1.100	1.049|1.153	< 0.001	***	Implant types	1.044	1.007|1.083	0.018	*
Incision-suture time	0.999	0.997|1.001	0.408	N.S.	Incision-suture time	0.999	0.998|1.001	0.646	N.S.
Lockdownstatus	0.997	0.959|1.037	0.891	N.S.	Lockdownstatus	1.000	0.972|1.033	0.887	N.S.
R^2^	0.303				R^2^	0.196			

Men					Women				
Total costs per patient	Coefficient	CI 95%	p-Value		Total costs per patient	Coefficient	CI 95%	p-Value	
Intercept	4285	3258|5642	< 0.001	***	Intercept	4614	3787|5623	< 0.001	***
Preoperative anemia	0.985	0.931|1.043	0.602	N.S.	Preoperative anemia	1,035	1.103|1.087	0.159	N.S.
EQ-VAS	1.000	0.999|1.001	0.346	N.S.	EQ-VAS	0.999	0.998|0.999	0.031	*
Age	1.002	0.999|1.005	0.086	N.S.	Age	1.002	1.000|1.001	0.046	*
BMI	1.003	0.998|1.008	0.274	N.S.	BMI	1.002	0.995|1.054	0.185	N.S.
Insurance status	0.982	0.927|1.040	0.531	N.S.	Insurance status	1.002	0.953|1.054	0.936	N.S.
ASA Score	1.026	0.985|1.069	0.205	N.S.	ASA Score	1.024	0.986|1.064	0.214	N.S.
Menendez Index	0.999	0.973|1.025	0.916	N.S.	Menendez Index	1.017	0.996|1.038	0.109	N.S.
TRX ia	1.076	1.027|1.127	0.003	**	TRX ia	1.057	1.019|1.095	0.003	**
Implant types	1.069	1.044|1.095	< 0.001	***	Implant types	1.054	1.035|1.074	<0.001	***
Incision-suture time	1.002	1.000|1.003	0.004	**	Incision-suture time	1.002	1.001|1.003	<0.001	***
Lockdownstatus	0.995	0.975|1.015	0.630	N.S.	Lockdownstatus	1.011	0.994|1.027	0.203	N.S.
R^2^	0.341				R^2^	0.325			

Hemoglobin in g/dl, CI = confidence intervall, significance levels *<0.05, **<0.01, ***<0.001, R^2^ = Mc Fadden R^2^

### Results postoperative Hb values

For men, the Hb loss between the preoperative Hb and the day before discharge was highly significantly associated with general ward costs and total costs. The general ward costs were reduced by 5.7% (approximately 161 Euros) with 1 g/dl less loss in Hb. The reductions for total costs were 3.7% (approximately 292 Euros) for 1 g/dl less loss in Hb. For women, the Hb on day 2 postoperatively was decisive, but only slightly significant for total costs (p < 0.05) and moderately significant for general ward costs (p < 0.01). General ward costs decreased by 2.6% (approximately 79 Euros) and total costs decreased by 1.8% (approximately 144 Euros) with 1 g/dl increased Hb. (Table 3) All models for postoperative Hb values with its pseudo R^2^ and AIC are shown in Table B (Supplementary material).

**Table 3:** Results postoperative Hb values

Men					Women				
General ward costs per patient	Coefficient	CI 95%	p-Value		General ward costs per patient	Coefficient	CI 95%	p-Value	
Intercept	782	468|1311	< 0.001	***	Intercept	2289	1674|3782	< 0.001	***
Hemoglobin loss (discharge-preoperative)	0.943	0.915|0.973	< 0.001	***	Hemoglobin day 2 postoperative	0.974	0.950|1.000	0.044	*
EQ-VAS	1.000	0.999|1.002	0.848	N.S.	EQ-VAS	0.999	0.997|1.000	0.031	*
Age	1.008	1.004|1.016	<0.001	***	Age	1.004	1.000|1.007	0.026	*
BMI	1.006	0.997|1.016	0.191	N.S.	BMI	1.005	1.000|1.010	0.091	N.S.
Insurance status	0.986	0.889|1.097	0.800	N.S.	Insurance status	1.064	0.966|1.174	0.212	N.S.
ASA Score	1.032	0.958|0.899	0.397	N.S.	ASA Score	1.037	0.964|1.115	0.330	N.S.
Menendez Index	1.039	0.991|1.090	0.115	N.S.	Menendez Index	1.028	0.989|1.068	0.172	N.S.
TRX ia	1.236	1.130|1.350	< 0.001	***	TRX ia	1.125	1.049|1.063	0.001	**
Implant types	1.063	1.014|1.115	0.010	**	Implant types	1.021	0.980|1.001	0.314	N.S.
Incision-suture time	0.998	0.996|1.000	0.124	N.S.	Incision-suture time	1.000	0.998|1.001	0.638	N.S.
Lockdownstatus	1.003	0.966|0.960	0.874	N.S.	Lockdownstatus	1.000	0.970|0.970	0.992	N.S.
R^2^	0.368				R^2^	0.177			

Men					Women				
Total costs per patient	Coefficient	CI 95%	p-Value		Total costs per patient	Coefficient	CI 95%	p-Value	
Intercept	3868	3013|4984	< 0.001	***	Intercept	5829	4381|7507	< 0.001	***
Hemoglobin loss (discharge-preoperative)	0.963	0.949|0.977	< 0.001	***	Hemoglobin day 2 postoperative	0.982	0.969|0.997	0.008	**
EQ-VAS	1.000	0.999|1.000	0.504	N.S.	EQ-VAS	0.999	0.999|1.000	0.048	*
Age	1.003	1.000|1.005	0.015	*	Age	1.002	1.000|1.003	0.055	N.S.
BMI	1.004	0.999|1.009	0.106	N.S.	BMI	1.002	1.000|1.005	0.089	N.S.
Insurance status	1.000	0.936|1.039	0.595	N.S.	Insurance status	1,007	0.956|1.056	0.784	N.S.
ASA Score	1.013	0.976|1.051	0.489	N.S.	ASA Score	1.017	0.986|1.061	0.363	N.S.
Menendez Index	1.000	0.976|1.023	0.972	N.S.	Menendez Index	1.016	0.997|1.038	0.123	N.S.
TRX ia	1.110	1.062|1.160	< 0.001	***	TRX ia	1.066	1.029|1.107	< 0.001	***
Implant types	1.047	1.023|1.071	< 0.001	***	Implant types	1.040	1.023|1.065	< 0.001	***
Incision-suture time	1.001	1.000|1.002	0.021	*	Incision-suture time	1.002	1.001|1.003	< 0.001	***
Lockdownstatus	0.998	0.980|1.017	0.864	N.S.	Lockdownstatus	1.011	0.995|0.974	0.158	N.S.
R^2^	0.445				R^2^	0.344			

Hemoglobin in g/dl, CI = confidence interval, significance levels *<0.05, **<0.01, ***<0.001, R^2^ = Mc Fadden (1977) R^2^

## Discussion

The main findings were the association of preoperative anemia for women with higher general ward costs and the association of distinct Hb values with total hospital costs. These findings were generally in line with previous findings about anemic patients, extended LOS, and subsequently higher costs. However, differences exist in sequelae of preoperative anemia between men and women. There were no associations for men with total hospital costs and general ward costs, whereas for women the general ward costs were associated with preoperative anemia due to increased LOS. This indicated that the preoperative Hb is not as important as the reductions for men and TKA. For women, the associations of the Hb on day 2 postoperatively was in line with the findings of previous literature, because it implies that the postoperative Hb depended on the preoperative Hb, already investigated by Cao et al. [4]. The natural process of Hb values with TKA and THA was evaluated by Cho et al. [6] with the focus on blood transfusions. Patients had the lowest Hb on day 3 postoperatively [6]. This is not directly in line with the findings of this study, as the mean Hb value was lowest on the day before discharge. It is worth mentioning that Cho et al. [6] investigated an inpatient period of 2 weeks, which is approximately 5–8 days longer than in Germany. Compared to several years ago, LOS has been reduced dramatically worldwide with TJR being even regularly performed in an outpatient set-up nowadays [10]. Regarding TRX, a recent review on blood management revealed reduced blood transfusion with TRX and therefore less costs [17]. TRX was related to hospital costs for both genders confirming its importance in TKA.

This study revealed that preoperative anemia and the association with general ward costs for TKA patients without blood transfusions was especially a problem for women, but not for men. This was mainly due to increased LOS for women. Whether men would have similar increasing LOS, the general ward costs would be significant, too. Since general ward costs consists to a large extent on personnel costs for nurses, less utilization of caring for patients could be beneficial for nursing shortages. Interestingly, Total hospital costs did not show any association with preoperative anemia, neither for men nor for women. This was due to other major costs components, such as implant types, which were comparable for anemic and non-anemic patients. In sum, cost containment with a correction of anemia may be feasible only for women for general ward costs, because the change in Hb is medically not easy to increase during the hospital stay. Although, for healthcare policy the findings of this study may be a starting point for further research on whether Hb values or other laboratory values could be a screening parameter for high-cost patients, and thus a reason for additional reimbursements for hospitals. The reimbursement system would have to differentiate between genders, because for women the levels of Hb were important and for men the reductions.

This study has several limitations. Due to the cross-sectional- and observational data only associations and no influence was analyzed. Generalizability is small, because only one hospital with a specific patient cohort (TKA due to OA without blood transfusions) was analyzed. There were only 21 women and 21 men with preoperative anemia. This is a relatively small number, hence there might be a bias, due to other reasons than anemia. Patients with blood transfusion were excluded, because the transfusion rate was approximately 1%, which was regarded to be too low for an analysis without a bias. Although the lockdown status for the COVID-19 pandemic was included as a dummy variable, a bias due to the pandemic cannot be excluded. TRX was generally applied for all patients with a consent on the medical information, despite not being approved for this indication. There are few contraindications where TRX was not given, including thrombosis, deep vein thrombosis and pulmonary embolism. Additionally, it was not given whether a patient had an apoplex, cardiac infarction or stent implantation within the last 12 months before surgery. This might cause a bias because patients were not randomly selected with and without TRX. All patients were treated with similar standards and therefore regarded to be comparable with respect to surgical approach and peri- and postoperative care. Nevertheless, there may be biases, due to different surgeons (for example use of tourniquet, drains and local infiltration analgesia). Moreover, the model selection was explorative, hence further confirmation is required. Costs were calculated according to the InEK standard, not distinguishing between direct and indirect costs. General ward costs and total hospital costs included indirect and direct costs. Whether solely the direct or indirect costs would be evaluated, the results might change.

## Conclusion

Preoperative anemia was a risk factor for high costs for women and general ward costs, but not for men. Total hospital costs decreased for specific Hb values, especially the difference between discharge and preoperative Hb for men, and day 2 postoperatively for women. Hence, preparation of patients and further development of reimbursement systems should consider both, preoperative anemia, especially for women, and postoperative Hb values. Monitoring Hb values appeared to be a goal congruence between medicine and financial management, because all significant results revealed cost reductions with improved Hb.

### Supplementary Information

Below is the link to the electronic supplementary material.

Supplementary file1 (PDF 35 KB)

## Data Availability

Data is not publicly available, due to privacy issues.
