# Supplementary material for: Associations of preoperative anemia and postoperative hemoglobin values with hospital costs in total knee arthroplasty (TKA)
Source: Arch Orthop Trauma Surg. 2023 Jun 12;143(11):6741–51. doi: 10.1007/s00402-023-04929-4 (PMC10258736; doi:10.1007/s00402-023-04929-4)
Supplement: Supplementary file 1 — Supplementary file1 (PDF 35 KB) [file 402_2023_4929_MOESM1_ESM.pdf]

Supplementary material

A. Sensitivity analyses

Methods sensitivity analyses

Preoperative anemia was included as a metric variable, instead of the binary variable to reveal possible challenges with the thresholds of anemia. Additionally, the gamma distribution was changed into a gaussian normal distribution to investigate the robustness of the models. GLMs with a gaussian normal distribution as link function are equal to ordinary least squares models, which were suggested for hospital cost analyses with relatively large samples [23].

Results sensitivity analyses

Including the preoperative Hb value instead of the binary variable of preoperative anemia for general ward costs indicated a robust model. This was also true for changing the gamma distribution to a normal Gaussian distribution, but the significance with the preoperative anemia was reduced from $p < 0.01$ to $p < 0.05$, and the magnitude of the coefficient was reduced by approximately 23 Euros. For men, the loss of Hb was still highly significant for total costs and general ward costs, merely the coefficient for the general ward costs was reduced by 13 Euros. For women, day 2 postoperatively had nearly the same coefficient with normal Gaussian distribution (only 3 Euros less with the same significance level), whereas the association with general ward costs vanished. In sum, the association of preoperative anemia with general ward costs for women was robust as well as day 2 postoperatively for women and total costs. For men, the Hb loss was robust for both, total costs, and general ward costs.

Table A: Results sensitivity analyses

Normal ward costs per patient	Men			Women		
Hemoglobin values	Coefficient	R^2	AIC	Coefficient	R^2	AIC
Preoperative	N.S.	0.308	2313.9	108.9 **	0.187	3034.5
Preoperative anemia binary	N.S.	0.301	2315.2	403.6 *	0.193	3033.2
Day 1 postpoperative	N.S.	0.307	2316.1	N.S.	0.172	3040.2
Day 2 postoperative	N.S.	0.312	2314.9	N.S.	0.174	3039.6
Discharge	N.S.	0.323	2312.6	N.S.	0.171	3040.4
Hemoglobin loss (discharge-preoperative)	-147.9 ***	0.363	2303.5	N.S.	0.159	3043.2

Total costs per patient	Men			Women		
Hemoglobin values	Coefficient	R^2	AIC	Coefficient	R^2	AIC
Preoperative	N.S.	0.298	2435.9	127.8 *	0.245	3186.0
Preoperative anemia	N.S.	0.288	2438.0	N.S.	0.237	3187.8
Day 1 postpoperative	N.S.	0.331	2430.7	-136.4 *	0.335	3163.2
Day 2 postoperative	N.S.	0.333	2430.3	-140.8 **	0.341	3161.5
Discharge	-131.2 *	0.358	2424.8	N.S.	0.327	3165.7
Hemoglobin loss (discharge-preoperative)	-291.3 ***	0.429	2407.4	N.S.	0.316	3168.8

Hemoglobin in g/dl, CI = confidence intervall, significance levels * <0.05, **<0.01, *** <0.001, R^2 = Mc Fadden (1977) R^2

B. Model comparison different perioperative Hb values

Table B: Results for different perioperative Hb values

Normal ward costs per patient	Men			Women		
Hemoglobin values	Coefficient	R^2	AIC	Coefficient	R^2	AIC
Day 1 postpoperative	N.S.	0.303	2315.1	N.S.	0.173	3033.4
Day 2 postoperative	N.S.	0.308	2314.2	**0.974 ***	**0.177**	**3032.6**
Discharge	N.S.	0.319	2311.8	N.S.	0.173	3033.5
Hemoglobin loss (discharge-preoperative)	**0.943 *****	**0.368**	**2300.7**	N.S.	0.159	3036.7

Total costs per patient	Men			Women		
Hemoglobin values	Coefficient	R^2	AIC	Coefficient	R^2	AIC
Day 1 postpoperative	N.S.	0.340	2423.4	0.983 *	0.337	3154.3
Day 2 postoperative	N.S.	0.342	2423.0	**0.982 ****	**0.344**	**3152.4**
Discharge	0.984 *	0.366	2417.3	N.S.	0.329	3156.7
Hemoglobin loss (discharge-preoperative)	**0.963 *****	**0.445**	**2397.7**	N.S.	0.312	3160.1

Hemoglobin in g/dl, CI = confidence intervall, significance levels * <0.05, **<0.01, *** <0.001, R^2 = Mc Fadden (1977) R^2
